# A General Synthesis Method for Patterning PEDOT toward Wearable Electronics and Bioelectronics

**DOI:** 10.34133/research.0383

**Published:** 2024-05-22

**Authors:** Wanke Cheng, Zihao Zheng, Xiaona Li, Ying Zhu, Suqing Zeng, Dawei Zhao, Haipeng Yu

**Affiliations:** ^1^ Key Laboratory of Bio-based Material Science and Technology of Ministry of Education, Northeast Forestry University, Harbin, China.; ^2^ Key Laboratory on Resources Chemicals and Materials of Ministry of Education, Shenyang University of Chemical Technology, Shenyang, China.

## Abstract

The conductive polymer poly-3,4-ethylenedioxythiophene (PEDOT), recognized for its superior electrical conductivity and biocompatibility, has become an attractive material for developing wearable technologies and bioelectronics. Nevertheless, the complexities associated with PEDOT’s patterning synthesis on diverse substrates persist despite recent technological progress. In this study, we introduce a novel deep eutectic solvent (DES)-induced vapor phase polymerization technique, facilitating nonrestrictive patterning polymerization of PEDOT across diverse substrates. By controlling the quantity of DES adsorbed per unit area on the substrates, PEDOT can be effectively patternized on cellulose, wood, plastic, glass, and even hydrogels. The resultant patterned PEDOT exhibits numerous benefits, such as an impressive electronic conductivity of 282 S·m^−1^, a high specific surface area of 5.29 m^2^·g^−1^, and an extensive electrochemical stability range from −1.4 to 2.4 V in a phosphate-buffered saline. To underscore the practicality and diverse applications of this DES-induced approach, we present multiple examples emphasizing its integration into self-supporting flexible electrodes, neuroelectrode interfaces, and precision circuit repair methodologies.

## Introduction

Poly-3,4-ethylenedioxythiophene (PEDOT) has garnered substantial interest because of its exceptional stability, biocompatibility, and unique ionic–electronic conductivity [1]. These qualities position PEDOT as a pivotal material for applications within energy storage [2,3], thermoelectric conversion [4–6], and biomedical electronics [7,8]. Extensive researches have been devoted toward refining the synthetic methods for PEDOT, with vapor phase polymerization (VPP), oxidative chemical vapor deposition (oCVD) [9–12], and electrochemical polymerization emerging as prominent techniques [13,14]. These methods have primarily facilitated the deposition of PEDOT particles and films onto flat substrates, such as silicon wafers or conductive glass. However, these approaches often encounter limitations due to electrode size, electrolyte concentration, or the need for additional vacuum and high-temperature conditions (exceeding 100 °C), which consequently lead to complex and expansive waste processing steps. Moreover, the adaptability of these methods to substrates—crucial for the creation of wearable PEDOT-based electronics—remains a challenge.

Deep eutectic solvents (DESs) represent a distinctive category of fluids where ions and molecules coexist, offering a tunable platform for material synthesis due to their structural designability [15]. A particular DES system comprising acetamide as a hydrogen bond (H-bond) donor and ferric chloride hexahydrate (FeCl_3_·6H_2_O) as an H-bond acceptor has been utilized to fabricate porous PEDOT [16]. In this system, the FeCl_3_·6H_2_O acts as a catalyst to initiate PEDOT polymerization, while acetamide, through H-bonding interactions with PEDOT, guides the epitaxial growth and assembly of PEDOT molecules. However, synthesizing PEDOT in the DES liquid phase poses challenges similar to those of solvent-based systems, such as uncontrolled polymerization behaviors and environmental contamination due to pollutant emission. Furthermore, the DES liquid-phase polymerization is less conducive to the deposition of patterned PEDOT on substrates in contrast to VPP and oCVD techniques.

In this work, we introduce a DES-induced VPP approach for the tailored, patterned deposition of PEDOT on diverse substrates. This novel DES composition, combining FeCl_3_·6H_2_O and urea, exhibits a strong affinity for a range of substrate materials, including wood, cellulose, plastic, and hydrogel, enabling direct writing or printing for pattern creation. Subsequently, the room-temperature volatilization of the 3,4-ethylenedioxythiophene (EDOT) monomer allows for PEDOT to undergo patterned polymerization with precision on these substrates—a capability not achievable with traditional methods. The DES-induced VPP process results in PEDOT patterns with a resolution of 200 to 400 μm, while also maintaining solid interfacial stability. Leveraging these attributes, we successfully apply the DES-induced VPP to the fabrication of flexible supercapacitors (SCs), circuit repair, and the construction of neural electrodes.

## Results

### Method and mechanism for patterning PEDOT

**Fig. 1.**
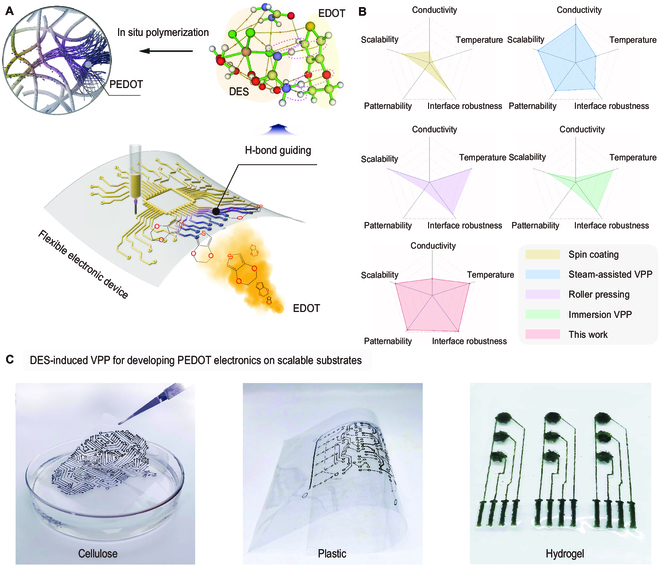
The DES-induced VPP approach for patterning deposition of PEDOT. (A) Schematic illustration of DES-induced VPP for the fabrication of flexible electronic devices. (B) Comparison of DES-induced VPP approach with conventional methods for the fabrication of patterning PEDOT. (C) Digital photographs of patterned PEDOT on cellulose paper, plastic (polyethylene terephthalate) film, and hydrogel by the DES-induced VPP.

The DES-induced VPP delineates a sophisticated and effective system for PEDOT polymerization. We first devise a DES composed of FeCl_3_·6H_2_O, serving as the necessary oxidant for polymerization, in combination with urea that functions as both a proton scavenger and a template for polymerization. This system is environmentally benign, operating efficiently at the modest temperature of 60 °C without the need for additional additives. The underlying mechanism involves H-bonding and ion complexation between FeCl_3_·6H_2_O and urea, which form the DES foundation. Notably, H-bonds play a pivotal role in linking the DES to the substrate and the EDOT to the DES. The EDOT monomers are efficiently captured and immobilized by the DES’s urea molecules through H-bonding. Subsequently, they are oxidized by Fe^3+^ ions into cationic radicals, which facilitate the in situ formation of PEDOT (as depicted in Fig. 1A and Fig. S1). As such, PEDOT polymerization and deposition occur exclusively at the DES-treated sites, enabling the precise patterning of PEDOT following the predefined configuration. Figure 1C and Fig. S2 depict photographic evidence of the patterned synthesis of PEDOT on assorted substrates, including cellulose materials, plastics, glass, and wooden sheets. Remarkably, the patterned PEDOT, when applied to the hydrogel with a water content exceeding 90 wt%, maintained its design and structural integrity; even upon immersion in water, it exhibited neither detachment nor degradation (Fig. S3). This result hints at a greater potential for DES-induced VPP in the production of bioelectronic materials and devices.

Extant methodologies for depositing PEDOT [17–19], such as casting [20], in situ polymerization [21], steam-assisted VPP [22], and 3-dimensional (3D) printing [23], are predicated on the substrate diffusion of oxidants of varying concentrations. These procedures necessitate multiple applications to achieve the requisite loading and electrical conductivity [24], which are both time-consuming and difficult to control. Compared with these methods, our DES-induced VPP offers substantial improvements for the patterning and precise fabrication of highly conductive PEDOT (refer to Fig. 1B and Table S1) [11,25], thereby streamlining the customization of electronic components suitable for the creation of a myriad of flexible devices. This outcome is unattainable through conventional methods such as oCVD or electrochemical polymerization.

### Controllable patterning of PEDOT

**Fig. 2.**
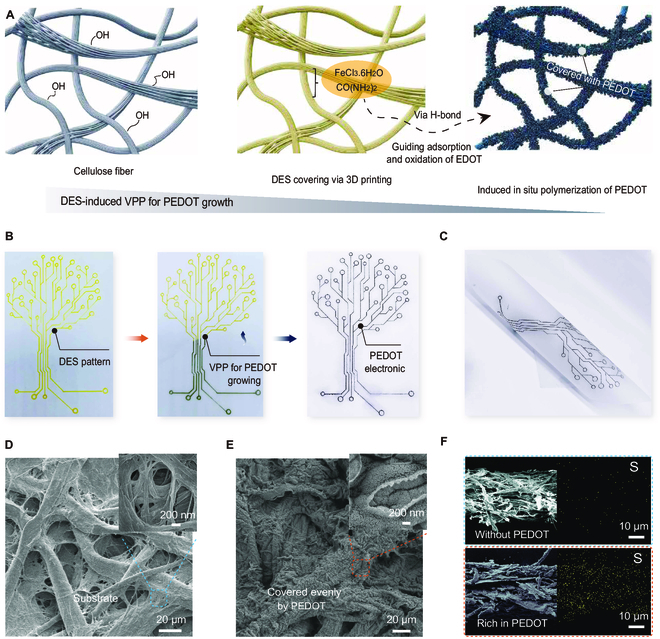
Patterning polymerization of PEDOT on cellulose substrate via DES-induced VPP approach. (A) Schematic illustration shows the in situ polymerization of PEDOT on cellulose substrate through free hydroxyl groups. (B) Photographs of patterning PEDOT on cellulosic substrate via the DES-induced VPP process. (C) Interfacial stability of patterning PEDOT with substrate for flexible electronics. (D and E) SEM image of cellulose substrate and that covered by PEDOT, respectively. (F) Comparison of cross-sectional mapping images of cellulosic substrate without PEDOT (top) and with rich PEDOT (bottom).

To showcase the delicacy and precision of patterned PEDOT structures achieved via the DES-induced VPP approach, we chose cellulose as a model substrate for patterning studies. Cellulose’s surface is rich in free hydroxyl groups (–OH) [26–29], which facilitate the creation of H-bonds with DES and EDOT, as illustrated in Fig. 2A. The substrate’s macroporous architecture, alongside its natural amphiphilic properties, offers copious adsorption sites for DES, thus ensuring a uniform deposition of DES over the cellulose substrate. This interaction gives rise to a quasi-solid interface replete with a dense H-bond network [30–33], inclusive of oxidants such as Fe^3+^ ions and proton scavengers like urea molecules. Following this, the EDOT monomers undergo oxidation by Fe^3+^ ions—a process propelled by the urea molecules—leading to in situ polymerization into conductive PEDOT (Fig. S4).

This DES-induced VPP approach enables precise control over both the placement and quantity of PEDOT on the substrate. By manipulating the velocity and number of cycles during direct writing or printing, the load and positioning of DES can be finely tailored. Consequently, PEDOT deposition occurs exclusively in areas with concurrent presence of DES and EDOT. For comparative purposes, the upper segment of the DES pattern retains its yellow hue when segregated from EDOT vapor, whereas areas exposed to EDOT are overlaid with black PEDOT in the predetermined pattern (Fig. 2B). Moreover, the deposited quantity of PEDOT can be regulated through temporal alterations to the constant concentration of EDOT vapor. The numerous H-bonds between PEDOT and cellulose substrate confer remarkable stability to the interface, preventing delamination even after extensive bending, depicted in Fig. 2C.

The morphological structures and characteristics of the PEDOT patterns formed through DES-induced VPP were explored with various microscopic techniques: scanning electron microscopy (SEM), transmission electron microscopy (TEM), and atomic force microscopy (AFM), with the results displayed in Fig. 2D to F. Comparing Fig. 2D and E, it is evident that the substrate is consistently coated with a layer of nanosphere-stacked PEDOT. The sulfur mapping distributions, indicative of the PEDOT structure, confirm the homogeneity of the PEDOT deposition on the substrate (Fig. 2F and Figs. S5 and S6). This uniform deposition underscores the unique capabilities of our DES-induced VPP approach. In contrast, a substitution of gaseous EDOT with its liquid-phase counterpart dripped onto a DES-treated cellulose substrate resulted in suboptimal PEDOT deposition, characterized by unevenness and local aggregations (Fig. S7).

### Characteristics and properties of patterning PEDOT

**Fig. 3.**
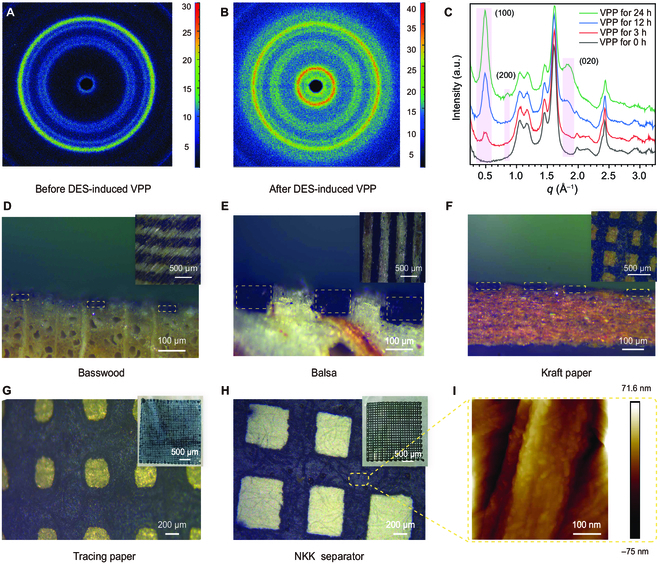
Crystalline structure and pattern fineness of patterning PEDOT via DES-induced VPP approach. (A and B) SAXS patterns of before and after DES-induced VPP polymerization of PEDOT. (C) SAXS profiles of patterning PEDOT with different polymerization time. a.u., arbitrary units. (D to F) Cross-sectional optical micrographs of the patterned PEDOT on textured substrates. Insets are surface micrographs. (G and H) Optical micrographs of the patterned PEDOT on smooth substrates. Insets are surface photographs. (I) AFM image of the patterned PEDOT on NKK separator.

Small-angle x-ray scattering (SAXS) was used to examine the spatial organization and crystallinity of patterned PEDOT films. Initial 2D SAXS analysis displays subdued pattern (Fig. 3A); however, after DES-induced VPP patterning, the substrate exhibits distinctly brightened and intensified PEDOT characteristic patterns (Fig. 3B). SAXS intensity profiles demonstrated that the unpatterned substrate exhibited minimal peaks at scattering vectors (*q*) of approximately 0.5, 0.85, and 1.85 Å^−1^, each corresponding to the (100), (200), and (020) crystal planes of PEDOT (Fig. 3C). With the advancement of the DES-induced VPP process, these peaks sharpened and grew in intensity. The growth in intensity may be due to the increase in the thickness of the crystalline layer of PEDOT, while the sharpening of peaks is indicative of a highly ordered and crystalline PEDOT structures.

The versatility of this DES-induced VPP approach is exemplified by its effective application to a variety of substrates. Beyond accommodating smooth surfaces like tracing paper, cellulose separator (NKK type) separator, glass, and plastic, it adeptly transfers precise patterns onto the textured surfaces of basswood, balsa wood, and kraft paper (Fig. 3D to I). The precision of patterned PEDOT deposition is essential, and the high resolution of our technique is evidenced by linewidth measurements conducted with SEM and AFM (Fig. 3I and Fig. S8). We achieve linewidths as narrow as approximately 200 μm, rivaling the precision of wearable electronics crafted by computerized embroidery [34], indicating that the DES-induced VPP approach can fabricate PEDOT patterns with exceptional detail and accuracy. This is because DES enriched with a high concentration of ions/molecules has a suitable surface tension that allows for limited diffusive spreading and necessary wettability of DES on a variety of substrates (Fig. S9).

### Mechanics, electrochemical performance, and deposit stability

**Fig. 4.**
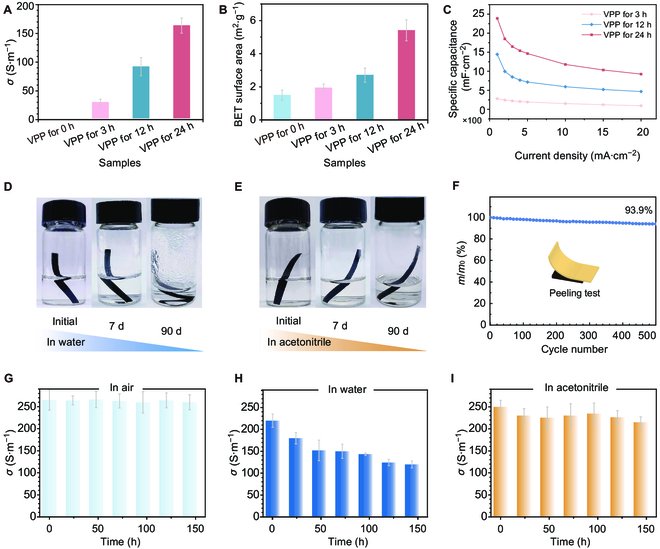
Performances of patterned PEDOT via DES-induced VPP approach. (A) Conductivity, (B) specific surface area, and (C) area-specific capacitance of patterned PEDOT via DES-induced VPP with different processing time. Digital photographs of the patterned PEDOT materials when soaked in (D) water and (E) acetonitrile for 7 and 90 d, respectively. (F) Mass retention of patterning PEDOT after tape tearing. Histogram of conductivity retention of the patterned PEDOT when being exposed in (G) air and soaked in (H) water and (I) acetonitrile, respectively.

We proceeded to assess the mechanical tensile properties, electrochemical capabilities, and interfacial robustness of the patterned PEDOT materials. The tensile strength of the patterned PEDOT-based materials reaches 18 MPa, as indicated in Fig. S10. Furthermore, the electrical conductivity of the PEDOT was measured at 282 S·m^−1^, which can be attributed to its highly crystalline structure (Fig. 4A and Table S2). This level of conductivity satisfies the requirements of a series of electronic applications, including SCs, electronic skins, and bioelectronics. Electrochemical evaluations of the patterned PEDOT (Figs. S11 and S12) reveal that with increased reaction time, more PEDOT was deposited per unit area on the substrate, resulting in a porous yet cohesive conductive network. The consequential specific surface area of PEDOT arrives at 5.29 m^2^·g^−1^ (Fig. 4B). Owing to its high conductivity, porous architecture, and substantial specific surface area, the patterned PEDOT demonstrates electrochemical performance that is both ideal and competitive.

The calculated area-specific capacitance (*C_a_*) values for PEDOT were derived from the galvanostatic charge–discharge (GCD) profile (Fig. 4C and Fig. S11). Notably, PEDOT synthesized on cellulose via DES-induced VPP for 24 h exhibits a remarkable *C_a_* of 2,220.8 mF·cm^−2^ at a current density of 1 mA·cm^−2^, along with commendable cycle stability (Fig. S12). This performance surpasses that of other composites such as cellulose nanofiber/PEDOT:polystyrene sulfonate (PSS) (854.4 mF·cm^−2^), PEDOT/bacterial cellulose (127.6 mF·cm^−2^), and carboxyl methyl cellulose/PEDOT (269 mF·cm^−2^) [35–37]. In addition, the thermogravimetric analysis indicates an ascendancy in the maximum degradation temperature of PEDOT—from 337.3 to 346.2 °C—with prolonged VPP durations (Fig. S10), suggesting an enhancement in thermal stability concurrent with processing time.

The interfacial stability between PEDOT and various substrates was rigorously examined through a series of experiments: a submersion dissolution test, a tape adhesion (peeling) test, and a conductivity retention test under different environmental conditions. Figure 4D and E depicts PEDOT strips submerged in water and acetonitrile, respectively. Digital photographic comparisons reveal no signs of PEDOT detachment after immersion periods of up to 90 d in water and in acetonitrile. The water remains clear, and the acetonitrile exhibits only a slight yellowing. No notable absorption peaks are detected in the ultraviolet absorption spectra of the liquids after immersion (Fig. S13), confirming the robust interfacial adhesion between PEDOT and a variety of substrates (Figs. S14 to S16). Furthermore, the tape peeling test corroborated this finding, with PEDOT retaining 94% of its mass after 500 cycles of adhesive application (Fig. 4F). Notably, the conductive stability testing revealed that PEDOT managed to maintain 60% to 95% of its conductivity in diverse environments of air, water, or acetonitrile, showcasing its resilience (Fig. 4G to I).

### Prospective applications

**Fig. 5.**
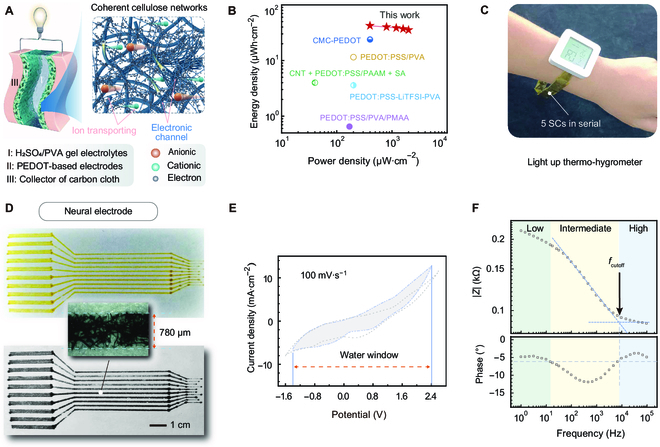
Application demonstrations of patterning PEDOT via DES-induced VPP. (A) Schematic illustration of a flexible SC assembled with PEDOT-based flexible electrodes. (B) Ragone diagram of SC devices. (C) Photographs of 5 SCs in series connection to light up a thermo-hygrometer. (D) Photographs and microscopic images of neural electrodes prepared by DES-induced VPP approach. (E) CV curves at 100 mV·s^−1^ and (F) Bode plot of single-channel of as-prepared neural electrodes in phosphate-buffered saline (PBS). CMC, carboxyl methyl cellulose; CNT, carbon nanotube; SA, sodium alginate; PAAM, polyacrylamide; LiTFSI, bis(trifluoromethane)sulfonimide lithium salt; PMAA, polymethacrylic acid.

The self-supporting PEDOT electrode, fabricated using the DES-induced VPP technique, boasts a porous structure, exceptional conductivity, and a large specific surface area. These characteristics enhance the wettability of electrolyte and increase the number of ion adsorption sites. In addition, the heightened conductivity of PEDOT facilitates rapid separation of electrons and holes, leading to improved interfacial charge transfer rates (Fig. 5A). Utilized in flexible SCs, the patterned PEDOT electrode exhibits remarkable electrochemical attributes (Fig. S17), including an area-specific capacitance of 489 mF·cm^−2^ at a current density of 1 mA·cm^−2^ and achieving an energy density of 42.02 μWh·cm^−2^ (Fig. 5B). These data precede those of most existing PEDOT-based SCs (Table S3) [38–41]. Demonstrating its applicability, a quintet of these flexible SCs was integrated in series within a wristwatch band, which served the dual purpose of powering a lighting circuit for electronic devices (Fig. 5C). These are a good demonstration of PEDOT’s potential in the field of wearable electronics.

The novel patterning approach applied to PEDOT also holds noteworthy promise for crafting neural electrodes. Distinct from previous PEDOT-based neural electrodes, the high conductivity of our material negated the need for a gold conductive layer [42]. Demonstrated in Fig. 5D, we have successfully fabricated a cellulosic neural electrode via our DES-induced VPP method. This technique enables PEDOT linewidths to be refined to several hundred micrometers, surpassing the precision of other reported patterned PEDOT materials prepared by oCVD [43]. This achievement is comparable to the 200 μm achieved through patterned PEDOT by metal electrode electrodeposition and inkjet printing [44,45]. Furthermore, the developed cellulosic neural electrode exhibited a stable voltage range of −1.4 to 2.4 V in PBS, detailed in Fig. 5E. The Bode plot analysis (Fig. 5F and Fig. S18) revealed a low contact resistance of 90 Ω and a solvent diffusion resistance at 1 kHz of approximately 113 Ω, vastly inferior to that observed in Au/bacterial cellulose electrodes of 55.6 kΩ [46]. This marked reduction in impedance suggests that our neural electrode is highly conducive for recording neural activity. In addition, an intriguing application of the DES-induced VPP approach was demonstrated for the in situ repair of damaged circuit boards (Fig. S19). While there is potential for enhancing conductivity further, this method facilitates rapid and straightforward restoration of electrical functionality.

## Conclusion

In this study, we systematically investigated a novel DES-induced VPP approach for controlled deposition and patterning of PEDOT. Utilizing this DES-induced VPP process at temperatures ranging from 25 to 60 °C, in conjunction with direct-write printing techniques, we successfully deposited PEDOT on a diverse array of substrate materials. The resultant PEDOT films exhibited superior interfacial adhesion and showcased electrochemical properties that are highly desirable. Remarkably, this method allowed for the precise patterning of PEDOT, consistently resulting in microscale resolution across a broad spectrum of substrates. This spectrum was not limited to, but included, adaptable materials such as cellulose, various plastics, wood, glass, and even hydrogels, illustrating the versatility of this method in accommodating different surface profiles and compositions. Such precision is crucial for the fabrication of complex curved electronic devices that demand intricate pattern detail.

The inherent simplicity of our DES-induced VPP approach, coupled with its versatility and scalability, markedly enhances its practicality for broader application. These features, allied with the high-resolution patterning capability, create consequential opportunities for use in the production of self-powered flexible electrodes, high-fidelity neuroelectrode interfaces, and the meticulous repair of electronic circuits requiring exacting detail and structural integrity. Collectively, these attributes position DES-induced VPP as a promising technology with the potential to make a momentous impact in the fields of wearable electronics and sophisticated bioelectronic devices.

## Materials and Methods

### Chemicals

The monomer EDOT (99%, Aladdin), FeCl_3_·6H_2_O (AR, 99%, Aladdin), urea (AR, Guangfu), methanol (AR, Fuyu), acetonitrile (AR, Fuyu), poly(vinyl alcohol) 1799 (PVA) [alcoholysis degree, 98% to 99% (mol/mol)], hydrochloric acid (HCl, 30%), sulfuric acid (H_2_SO_4_, 98%), and PBS (pH 7.3 to 7.5, 1×, Aladdin). All chemicals were used as received without any further purification.

### Preparation of DES

In a standard synthesis of DES, a predefined molar ratio of FeCl_3_·6H_2_O to urea was 1:2 measured into a single glass flask. The resultant mixture was then subjected to magnetic stirring at 60 °C, which, after approximately 30 min, yielded the desired DES.

### Time-gradient preparation of patterning PEDOT by DES-induced VPP

Commercial cellulose filter papers, 175 μm in thickness, were precisely cut into squares measuring 2 cm by 2 cm. These squares were arranged on a pristine glass sheet to serve as substrates. Utilizing a plastic pipette, we applied a specially prepared DES to the substrate, ensuring that the cellulose pieces were thoroughly impregnated with the solution. Excess DES was carefully blotted away using additional filter papers, resulting in the treated material referred to as “DES@cellulose”. The DES@cellulose was then introduced to a polytetrafluoroethylene reactor along with 0.1 g of the EDOT monomer. The reactor was hermetically sealed and placed in an oven preheated to 60 °C. After a predetermined duration, the reactor was removed and allowed to cool in a fume hood. Subsequent to the reaction, the now-blackened paper sheets were washed with methanol repeatedly until the wash solution ran clear, indicating the removal of unreacted components. The papers were then air-dried within the fume hood. The resulting samples were systematically categorized on the basis of their respective exposure times to the VPP process, designated as VPP for 0 h, VPP for 3 h, through to VPP for 24 h, in increments of 3 h. This nomenclature enabled a clear reference point for analyzing the impact of reaction duration on the characteristics of the PEDOT-coated cellulose substrate.

### Patterning PEDOT on different substrates

A microelectronic printer (Scientific 3A model, Prtronic, Shanghai, China) operated in dispensing mode was used for designing and printing on various substrates. The printer featured a printing needle with an inner diameter of 0.16 mm. It was capable of operating within a pressure range of 0.5 to 60 psi, and the printing speed was adjustable between 1 and 6 mm·s^−1^. For printing on smooth substrates, including polyethylene terephthalate (PET), polyimide (PI), transfer paper, glass, A4 paper, and acrylonitrile butadiene styrene plastic (ABS), as well as kraft paper, a full plastic T-needle was utilized. In contrast, a specialized soft bristle brush needle was used for substrates with rougher surfaces such as fabric, filter paper, wood, and ethylene vinyl acetate copolymer (EVA). The substrate size selected for this study typically conformed to B5 dimensions (250 mm by 176 mm), aligning with the maximum printable area afforded by the microelectronic printer model in use. The designed pattern of DES combined with the EDOT monomer was then introduced into a polytetrafluoroethylene reactor, which was subsequently sealed and placed into an oven set at 25 °C for polymerization.

### Characterization methods of patterning PEDOT

In morphological characterization, SEM images were captured using a microscope (JSM-7500F, JEOL, Japan). TEM images were obtained with a microscope (JEM-2100, JEOL, Japan). The surface area analysis was conducted with a Brunauer–Emmett–Teller (BET) surface area analyzer (ASAP 2460, Micromeritics Instrument Co. Ltd., China). Prior to BET testing, samples were prepared by cutting them into the smallest possible size: The SAXS patterns were obtained using an SAXS structure analyzer (Xeuss 2.0, Xenocs, France) with a copper target and a light tube power of 30 W. The detector used was PILATUS 300K with a single pixel size of 0.172 mm. Mechanical performance was evaluated through tensile testing conducted with a universal mechanical testing machine (U60, Gotech, China) in stretching mode. Each group consisted of 5 samples measuring 20 cm by 0.5 cm by 0.172 to 0.275 mm, tested at a speed of 5 mm·min^−1^ at room temperature.

### Characterization of the electrochemical properties of patterning PEDOT

The conductivity was tested using a digital 4-probe tester (RTS-8, 4probes, Guangzhou). The chronoamperometry (CV), GCD, and electrochemical impedance spectroscopy (EIS) were performed with an electrochemical workstation (CHI 760E, Chenhua, Shanghai, China). The CV and GCD tests were carried out within a voltage range of −0.2 to 0.8 V, with scan rates of 10, 20, 50, 100, 200, and 500 mV·s^−1^. The EIS test spanned a frequency range from 10^5^ to 10^−2^ Hz, with an amplitude of 5 mV. In the 3-electrode system, a 1 M HCl aqueous solution served as the electrolyte, a Pt electrode (10 mm by 10 mm by 0.1 mm) functioned as the counter electrode, and an Ag/AgCl electrode was used as the reference electrode. The EIS data were analyzed using the Z view software to derive the fitted data and equivalent circuit diagrams. The area-specific capacitance of PEDOT electrodes in 3-electrode systems is calculated on the basis of the Eq. 1:

$$C_{a}=\frac{I\times \Delta t}{S\times \Delta V}$$ (1)

where *C_a_* is the area-specific capacitance (in millifarads per square centimeter), *I* is the discharge current (in milliamperes), Δ*t* is the discharge time (in seconds), Δ*V* is the potential window during the discharging (in volts), and *S* is the area of the PEDOT self-supporting electrodes (in square centimeters).

### Stability tests on patterning PEDOT

A thermogravimetric analyzer (STA 6000-SQ8, PE, USA) was utilized to assess the thermal stability of the PEDOT/cellulose sample in a nitrogen atmosphere, with a heating rate of 10 °C·min^−1^. The PEDOT/cellulose strips, measuring 4 cm by 0.5 cm, were then immersed in 10 ml of deionized water and 10 ml of acetonitrile to evaluate the dissolution stability of PEDOT. Three milliliters of each undiluted liquid was collected for ultraviolet absorption spectroscopy after 1 week with clean deionized water and acetonitrile, respectively serving as control samples. The samples were then sealed and left to stand for 3 months for observation. In addition, those patterning PEDOT samples printed on various substrates were also immersed in water for a duration of 2 d to assess pattern retention. To investigate the interfacial stability of deposited PEDOT on cellulose substrates, the sample surface underwent repeated taping with PI tape, and the resulting weight changes were recorded over 500 cycles. Furthermore, to assess the stability of PEDOT in various environments, the samples were placed in air, water, and acetonitrile, with conductivity measurements taken every 24 h. Each set of samples included 5 parallel samples.

### Electrochemical tests of patterning PEDOT-based SCs

Electrochemical analyses were conducted using a CHI760E electrochemical workstation (Chenhua Instruments, Shanghai, China), operating with a 2-electrode setup. A symmetric SC using PEDOT was constructed to form a sandwich-structured device. The flexible electrolyte used was a gel synthesized by dissolving 6 g of PVA together with 6 g of H_2_SO_4_ in 60 ml of deionized water, followed by continuous stirring at 90 °C for 1 h. Carbon cloth served as the current collector, and the device assembly was sealed with PI tape for encapsulation. The area-specific capacitance of PEDOT based SC in a 2-electrode system is calculated on the basis of the Eq. 2:

$$C_{\text{SC}}=\frac{2\times I\times ∆t}{S\times ∆V}$$ (2)

where *C*_SC_ is the area-specific capacitance (in millifarads per square centimeter), *I* is the discharge current (in milliamperes), Δ*t* is the discharge time (in seconds), Δ*V* is the potential window during the discharging (in volts), and *S* is the total electrode area (in square centimeters).

The energy density (*E*) and average power density (*P*) were calculated on the basis of the Eqs. 3 and 4:

$$E=\frac{C_{\mathrm{SC}}\times ∆\;V^{2}}{7.2}$$ (3)

where *E* is the energy density (in microwatt-hour per square centimeter), *C*_SC_ is the specific capacitance (in millifarads per square centimeter), and Δ*V* is the potential window during the discharging (in volts).

$$P=\frac{3,600E}{∆\;t}$$ (4)

where *P* is the power density (in microwatts per square centimeter), *E* is the energy density (in microwatt-hour per square centimeter), and Δ*t* is the discharge time (in seconds).

### Preparation and characterization of neural electrodes by DES-induced VPP

Patterns of neural electrodes were drawn on cellulose using DES as ink through the dispensing mode of a microelectronic printer (Scientific 3A model, Prtronic, Shanghai, China). Then, it was put into a 60 °C closed reactor with EDOT atmosphere for VPP. The prepared neural electrodes were rinsed with methanol until the filtrate was colorless and then air-dried in a fume hood.

The CV and EIS were performed with an electrochemical workstation (CHI 760E, Chenhua, Shanghai, China). The voltage range of CV is −1.4 to 2.4 V, the scan rate is 100 mV·s^−1^, the frequency range of EIS test is 10^5^ to 10^0^ Hz, and the amplitude is 5 mV. For the 3-electrode system, the electrolyte was 1 M PBS solution, the counter electrode was Pt electrode (10 mm by 10 mm by 0.1 mm), and Ag/AgCl electrode was used as reference electrode.

## Data Availability

All data supporting the findings of this study are available within the paper and its Supplementary Materials. In addition, the datasets generated or analyzed during this study are available from the corresponding author on reasonable request.

## Supplementary Materials

Supplementary 1 Figs. S1 to S19 Tables S1 to S3 References [47,48]
