# Supplementary material for: A General Synthesis Method for Patterning PEDOT toward Wearable Electronics and Bioelectronics
Source: Research (Wash D C). 2024 May 22;7:0383. doi: 10.34133/research.0383 (PMC11109514; doi:10.34133/research.0383)
Supplement: Supplementary 1 — Figs. S1 to S19 Tables S1 to S3 References [47,48] [file research.0383.f1.docx]

**A General Synthesis Method for Patterning PEDOT toward Wearable Electronics and Bioelectronics**

Wanke Cheng^1^, Zihao Zheng^1^, Xiaona Li^1^, Ying Zhu^1^, Suqing Zeng^1^, Dawei Zhao^1,2,^*, Haipeng Yu^1,^*

^1^ Key Laboratory of Bio-based Material Science and Technology of Ministry of Education, Northeast Forestry University, Harbin, 150040 China

^2^ Key Laboratory on Resources Chemicals and Materials of Ministry of Education, Shenyang University of Chemical Technology, Shenyang, 110142 China

* Corresponding author E-mail:

[yuhaipeng20000@nefu.edu.cn](mailto:yuhaipeng20000@nefu.edu.cn) (Haipeng Yu)

[syzhaodawei2014@nefu.edu.cn](mailto:syzhaodawei2014@nefu.edu.cn) (Dawei Zhao)


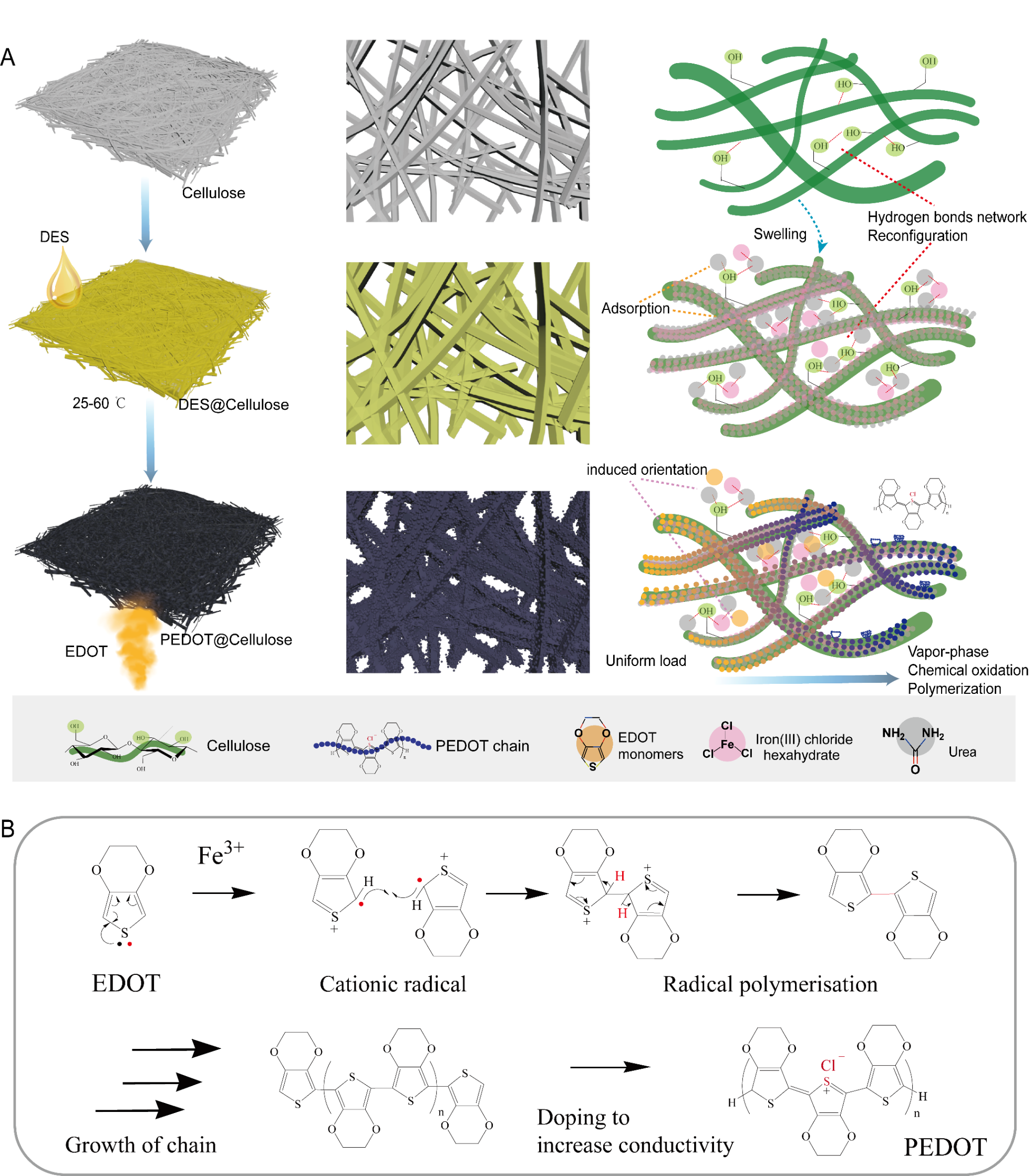


Fig. S1. DES-induced in situ polymerization for the patterning PEDOT. The role of H-bond and DES guidance in PEDOT polymerization and interfacial stabilization was illustrated taking cellulose substrates as an example. This mechanism is applicable to other substrates with -OH-rich surfaces that are prone to H-bond.


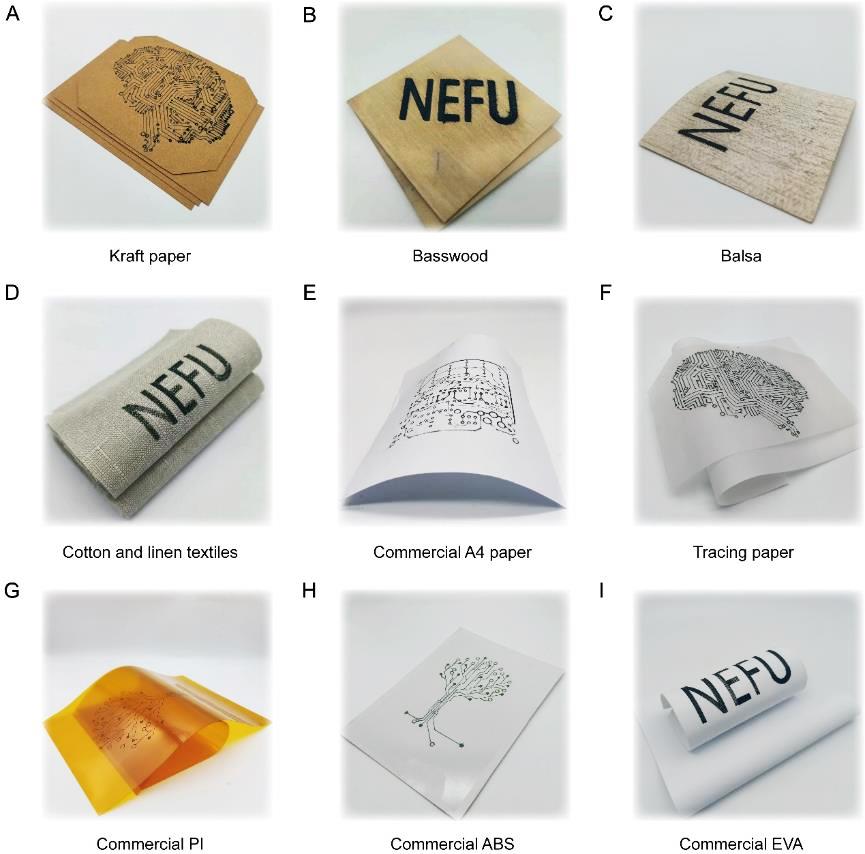


**Fig. S2.** Physical pictures of patterning PEDOT on different substrates by DES-induced VPP. A-F) Commercially available cellulose-based substrates: kraft paper (A), basswood (B), balsawood (C), cotton and linen fabric (D), printing paper (E) and tracing paper (F); G-I) Engineering plastic-based substrates: polyimide (G, PI), acrylonitrile butadiene styrene plastic (H, ABS) and ethylene vinyl acetate copolymer (I, EVA).


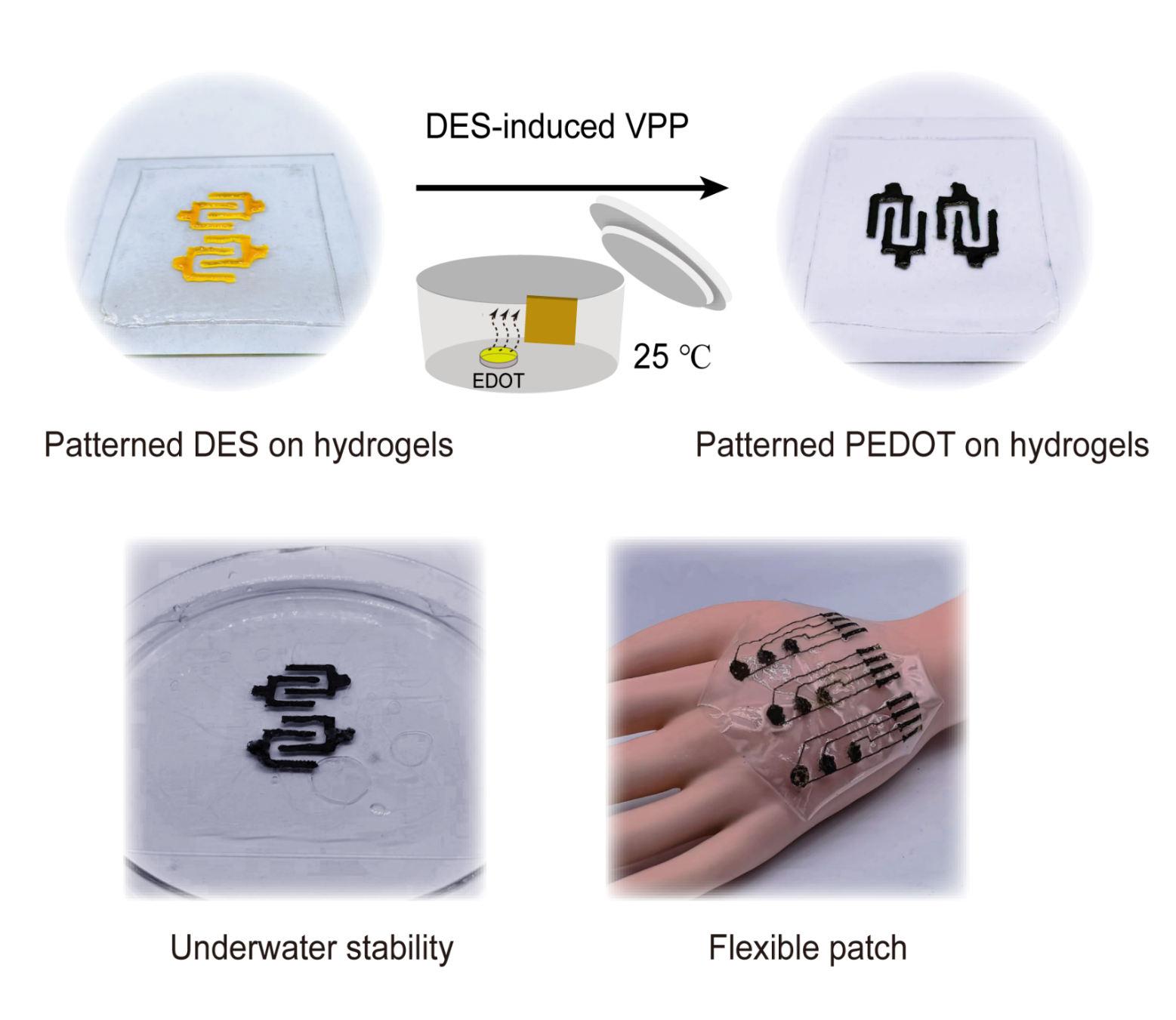


Fig. S3. Physical picture of controllable preparation, stability of the flexible PEDOT patch via DES-induced VPP. Patterned DES on polyvinyl alcohol (PVA) hydrogels and EDOT monomer was sealed into the PTFE reactor at room temperature. The obtained patterning PEDOT on hydrogel retained the flexibility of the substrate.

The precision of the patterns PEDOT on hydrogels was limited because it is really not possible to eliminate the diffusion of the DES components in water, since both ferric chloride and urea are extremely water-soluble molecules. Therefore, we used a rectangular pattern in our first attempts, as shown here, so that the diffusion overlap of the different print lines would be kept within limits. In order to print even finer lines, we will adopt some surface hardening strategies on a thick gel film to slow down the diffusion. And the deposition of PEDOT is started quickly after the DES pattern is printed. The gel is placed in a constant temperature and humidity chamber overnight, in order to balance the overall moisture.


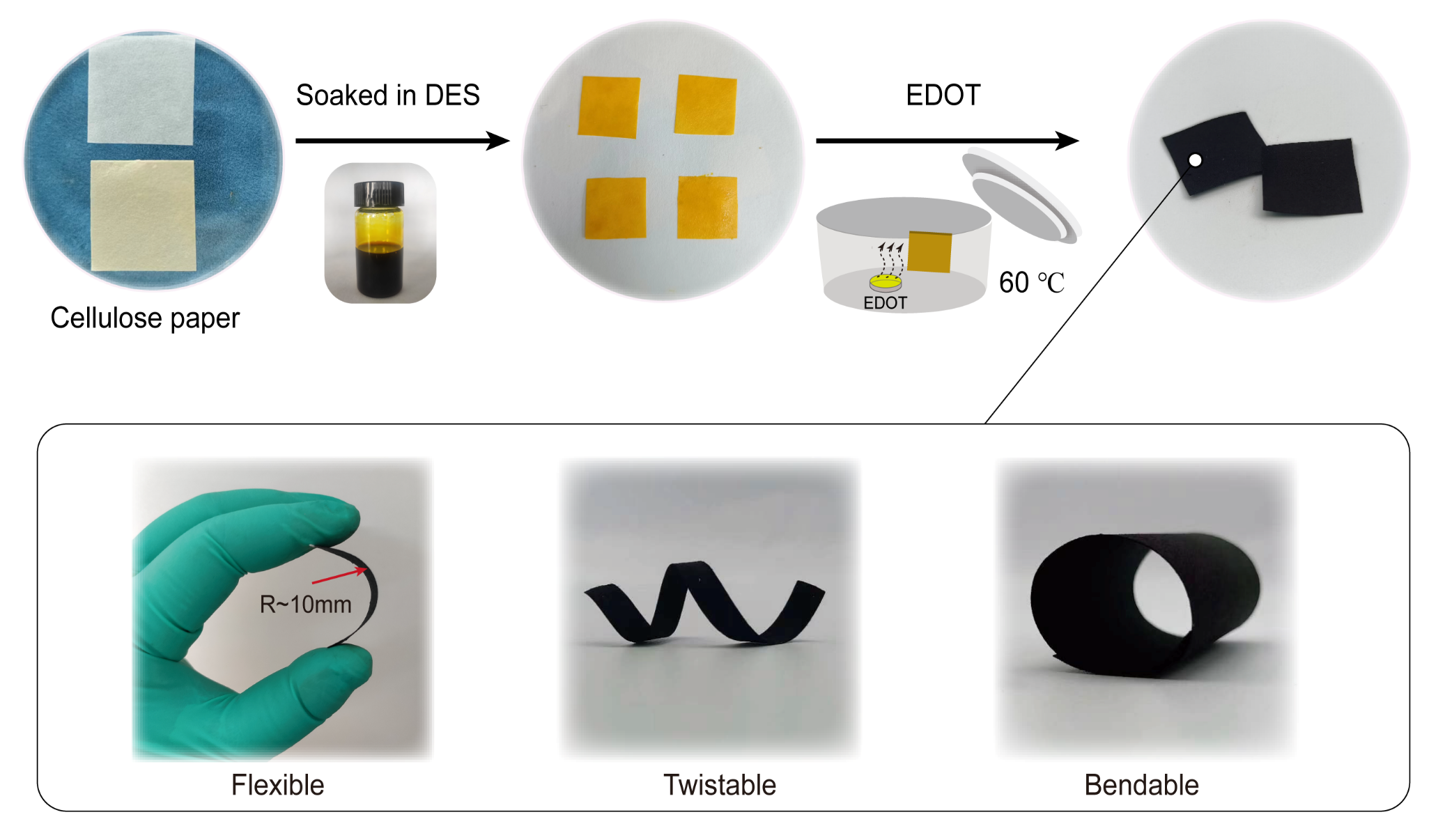


Fig. S4. Physical picture of controllable preparation, flexibility, and interfacial stability of the PEDOT materials via DES-induced VPP.


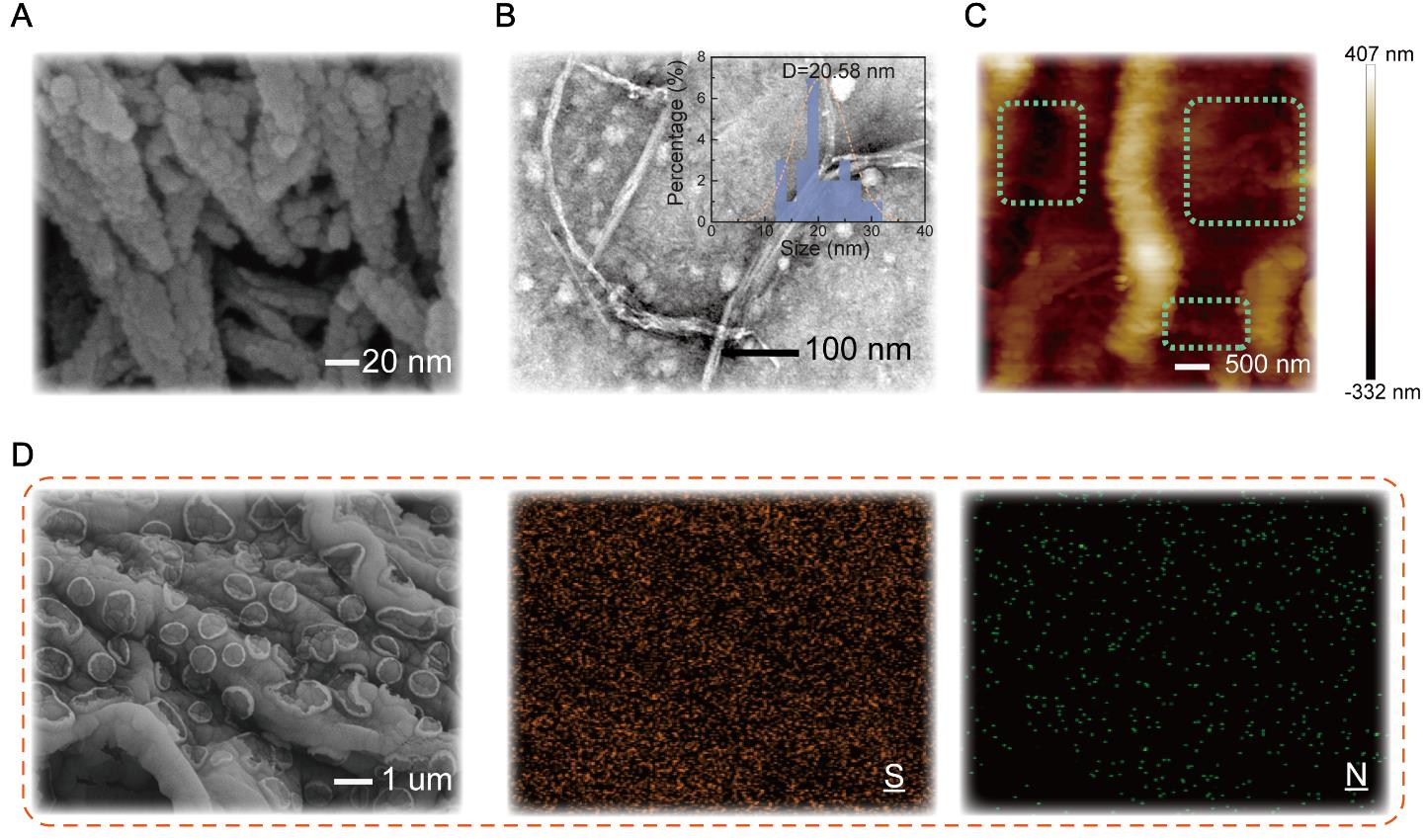


Fig. S5. Microscopic morphology of patterning PEDOT by DES-induced VPP. A-C) SEM images at high magnification (A), TEM images (B) and AFM images (C) of controllable preparation of PEDOT by DES-induced VPP. D) SEM image and mapping at low magnification of PEDOT loaded on substrate.


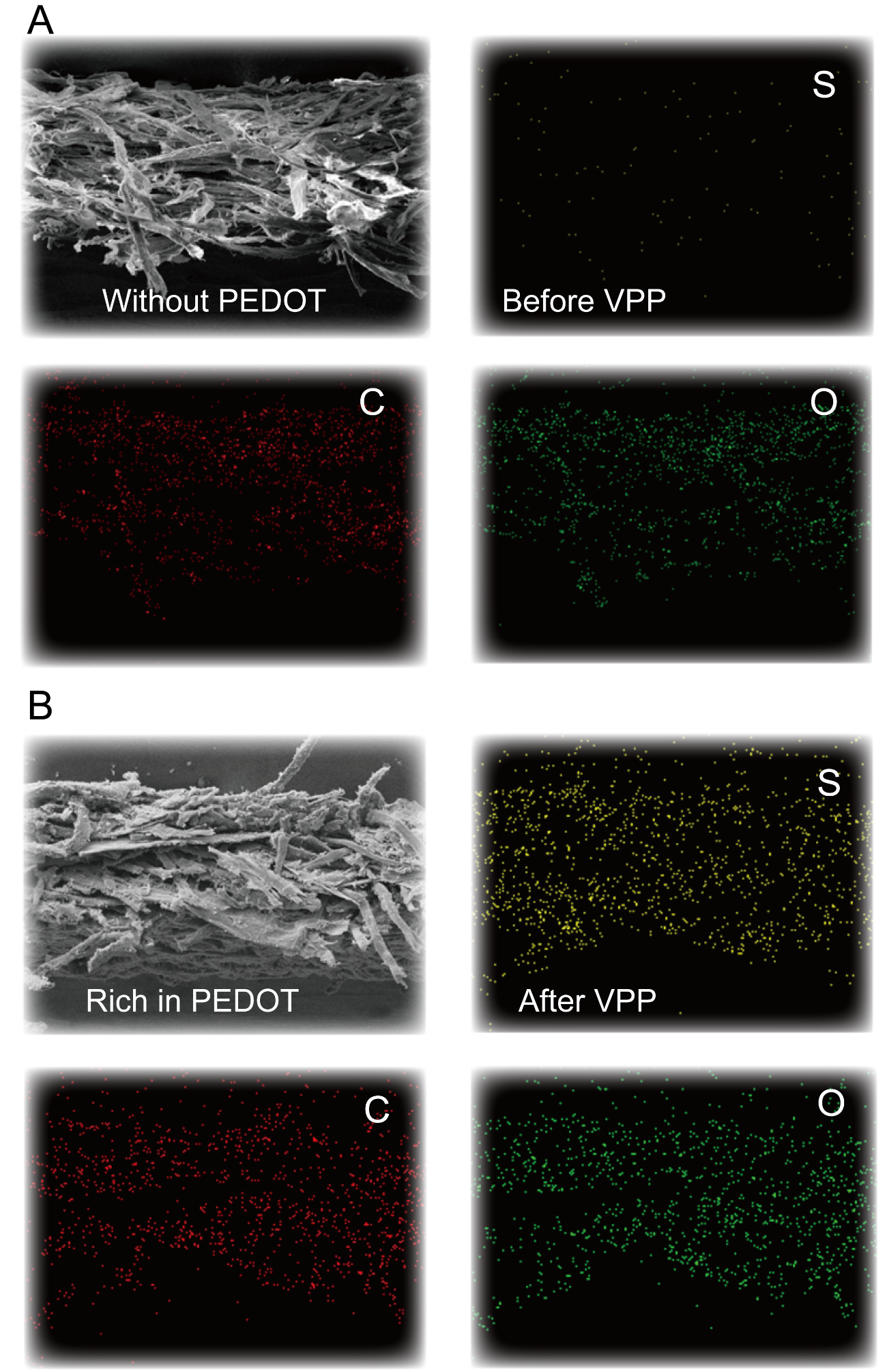


Fig. S6. SEM images at low magnification in cross section and corresponding mapping images before (A) and after (B) PEDOT loaded on cellulose substrate.


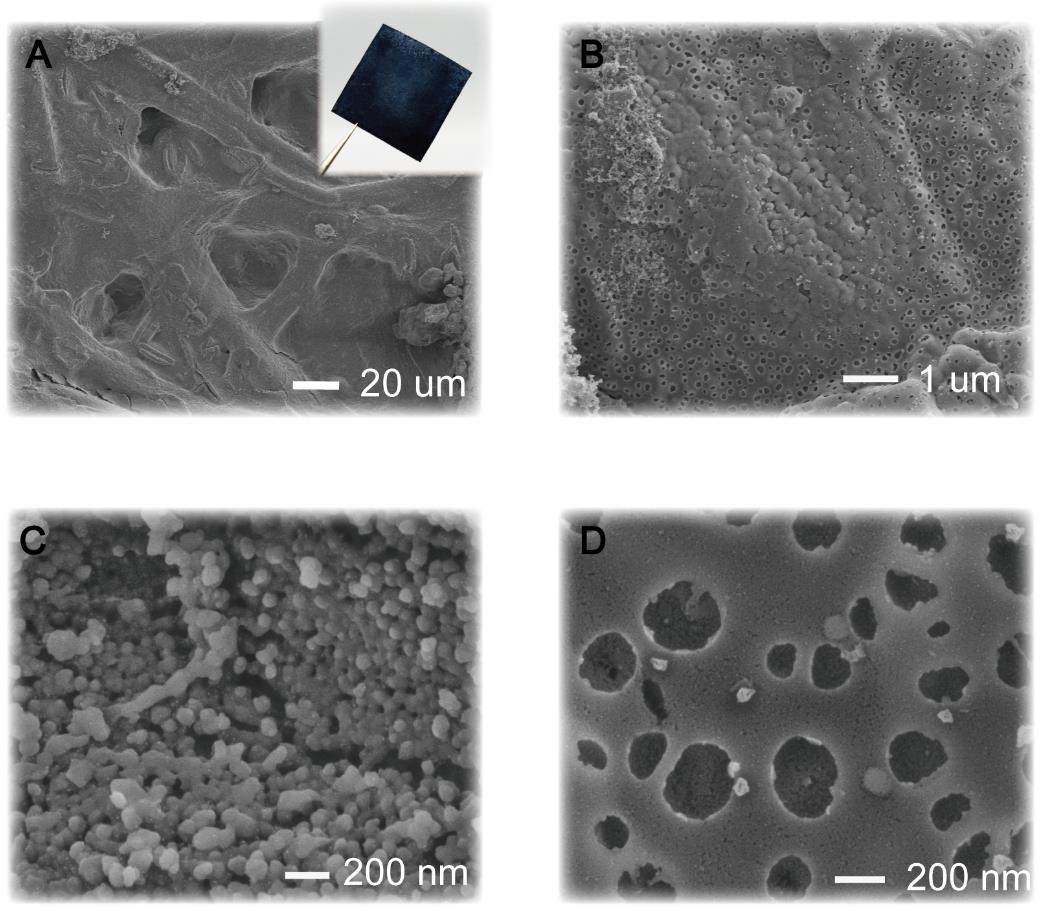


Fig. S7. Microscopic morphology of PEDOT on cellulose by DES-induced liquid phase polymerization. A) SEM image at low magnification of PEDOT loaded on substrate. The inset is a corresponding digital photo. B) SEM image at medium magnification of PEDOT loaded on substrate. C and D) SEM image at high magnification of PEDOT loaded on substrate in homogeneous PEDOT regions (C) and low DP PEDOT agglomeration regions (D), respectively.


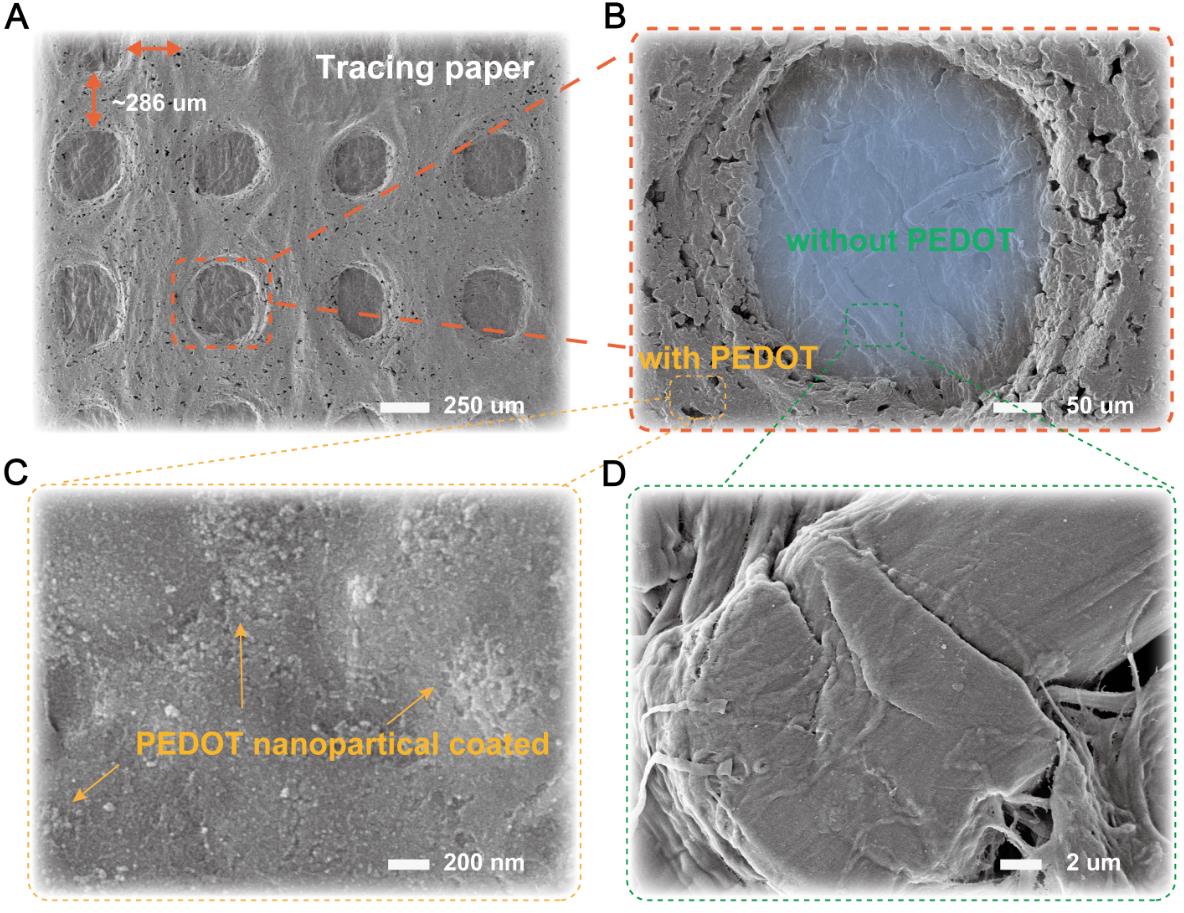


Fig. S8. Microscopic morphology of patterning PEDOT printed on tracing paper via DES-induced VPP method. A) SEM image at low magnification of PEDOT loaded on substrate. B) SEM image at medium magnification of PEDOT loaded on substrate. C) SEM image at high magnification of PEDOT loaded on substrate. D) SEM image at medium magnification of substrate which PEDOT unloaded on.


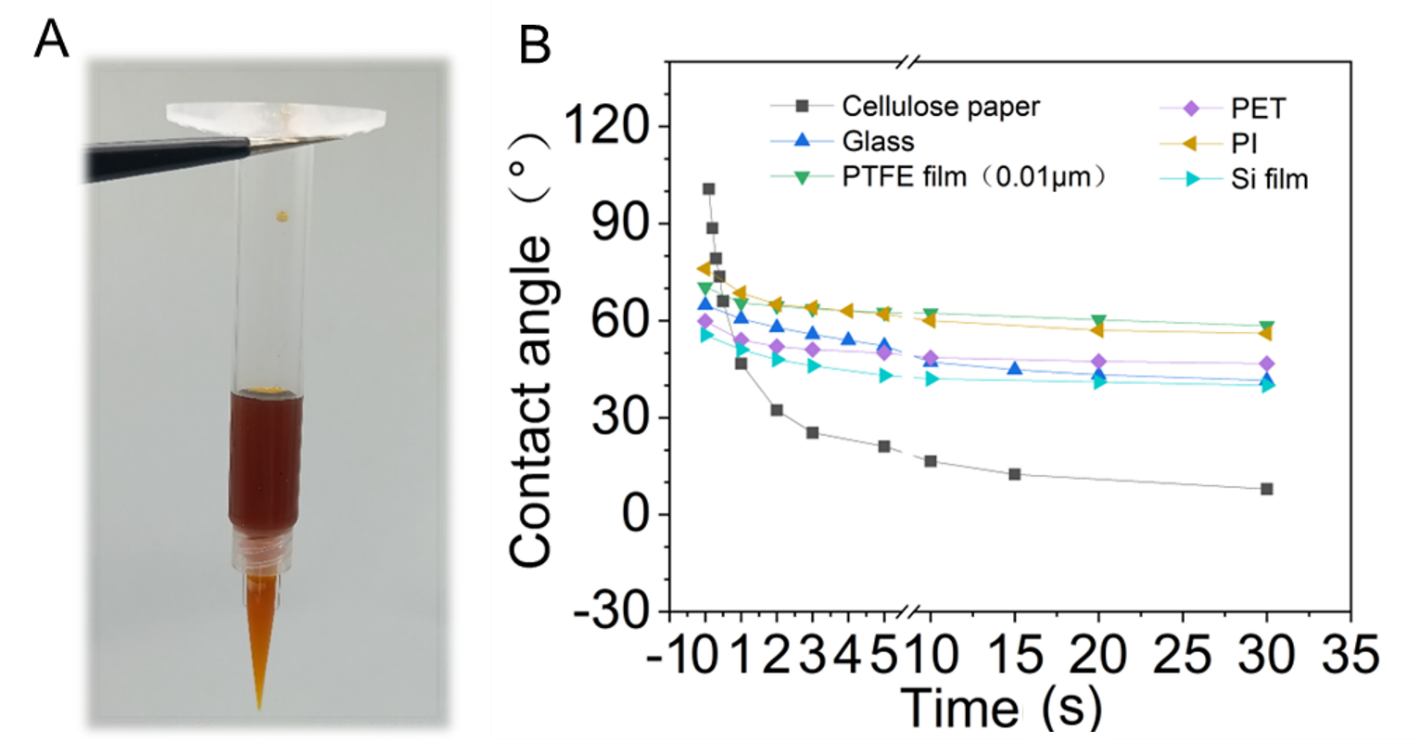


Fig. S9. Physical properties of DES. A) Physical photo of DES in a print ink capsule. B) Dynamic contact angle variation curves of DES on different substrates.

Because DES is a mixture of highly concentrated ionic/molecular compositions, it is much more viscous than commonly used oxidizer-solvent. So, it can be kept in a syringe with a certain size that does not drip when no additional pressure is applied, which can be smoothly extruded under pressure. This is an important feature that distinguishes it from other VPPs. DES is limited diffusion on the substrate making the printing accuracy not smaller to a few microns but confined to a few hundred microns.


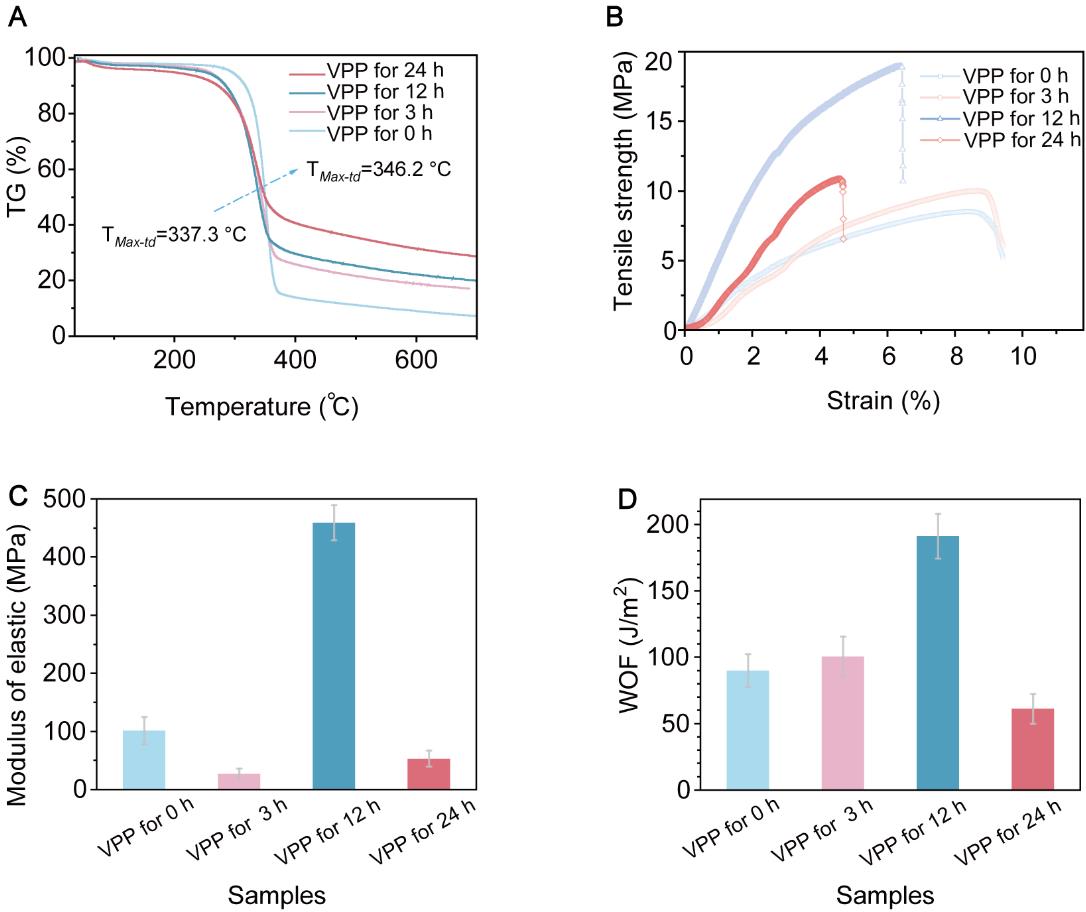


Fig. S10. Thermal stability and mechanical properties of patterning PEDOT. A-D) Comparison of thermogravimetric curves (A), tensile properties (B), elasticity modulus (C), and WOF energy values (D) of patterning PEDOT at different time gradient.


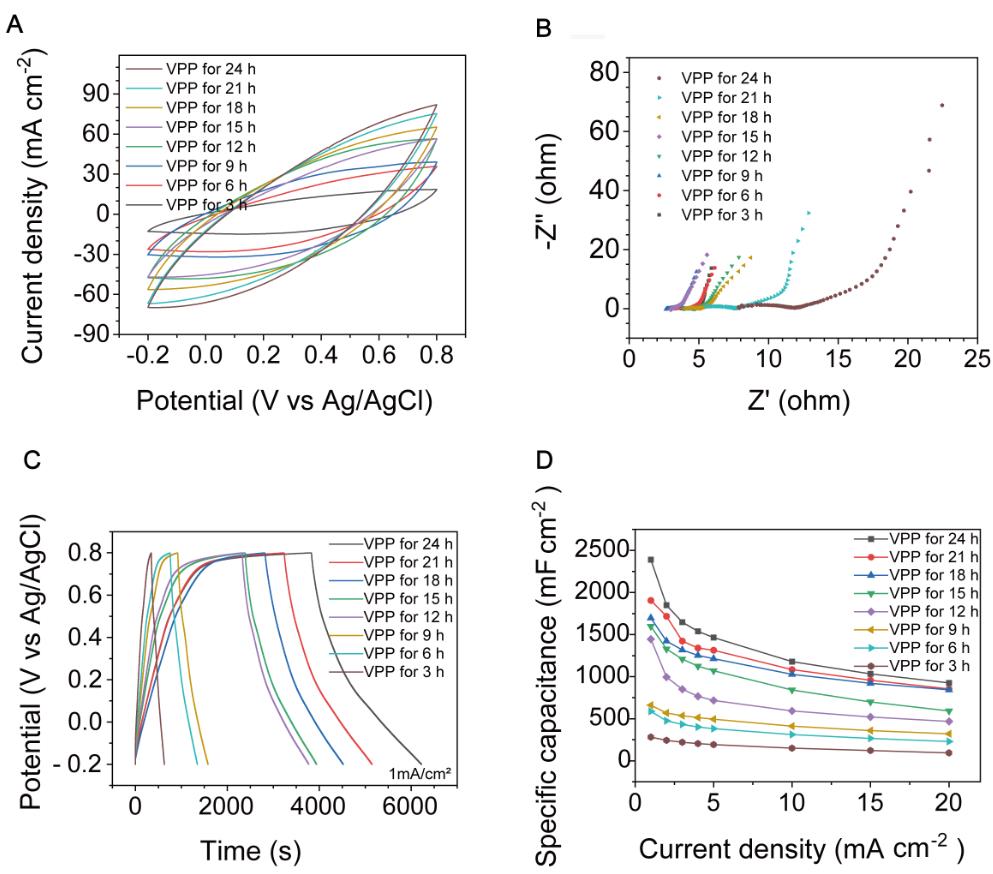


Fig. S11. Three-electrode electrochemical performance comparison of PEDOT based self-supporting electrode after VPP for different time. A) CV curves at 100 mV/s. B) Nyquist plots. C) GCD profiles at 1 mA/cm^2^. D) Plot of the area-specific capacitance calculated from the GCD profiles.


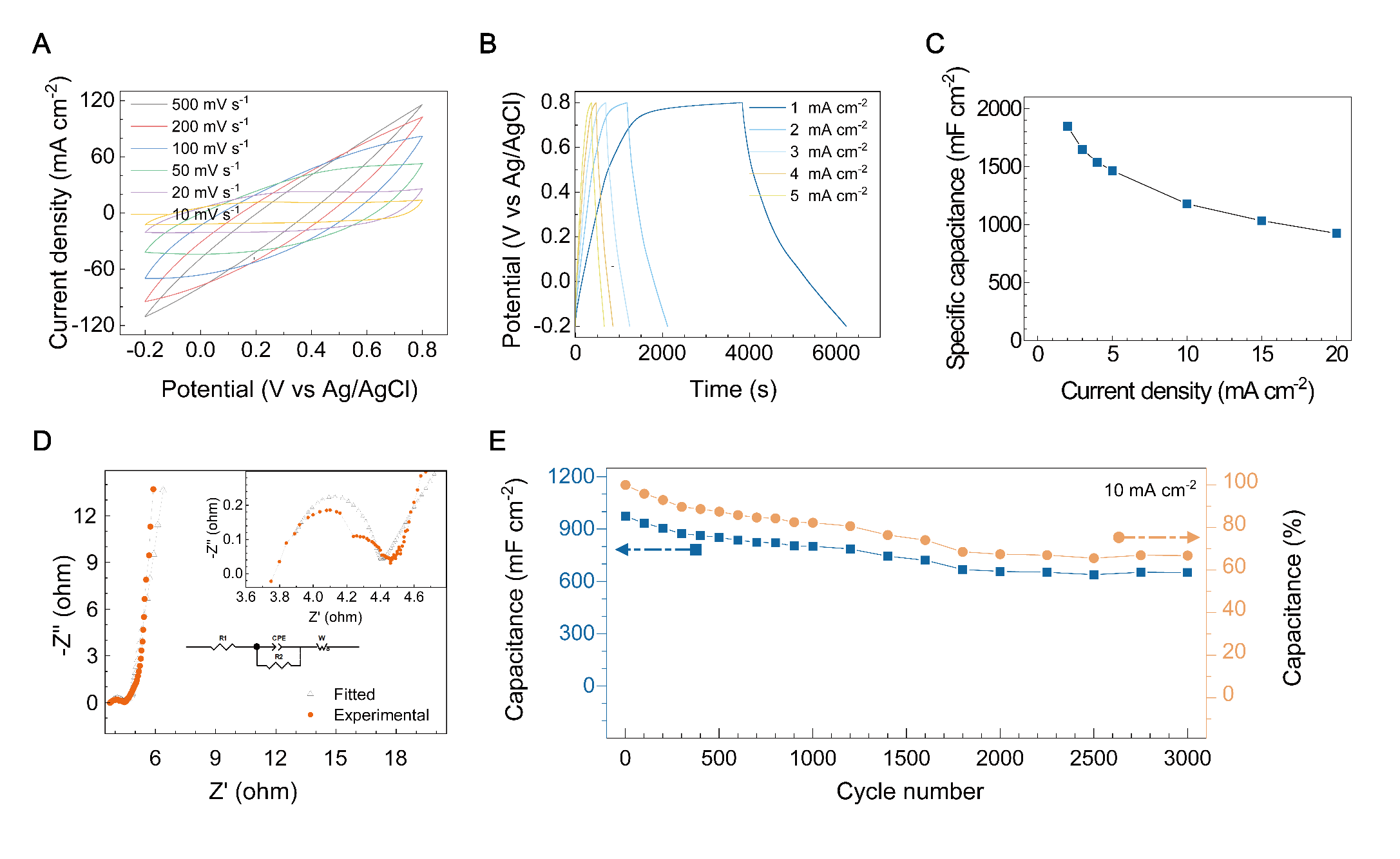


Fig. S12. Three-electrode electrochemical performance of patterning PEDOT self-supporting electrode of VPP for 24h. A) CV curves at different scan rates. B) GCD profiles at different current densities. C) Plot of the area-specific capacitance calculated from the GCD profiles. D) Nyquist plots. E) Charge and discharge stability at 10mA/cm^2^.


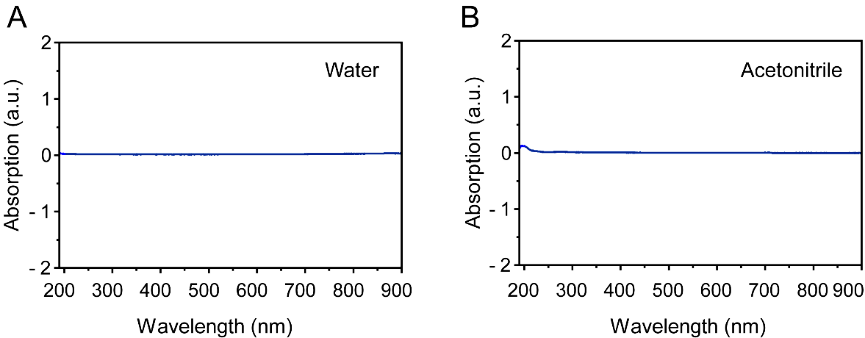


Fig. S13. Ultraviolet (UV) absorption spectra of the filtrates of patterning PEDOT soaked in water (A) and acetonitrile (B) for one week, respectively.


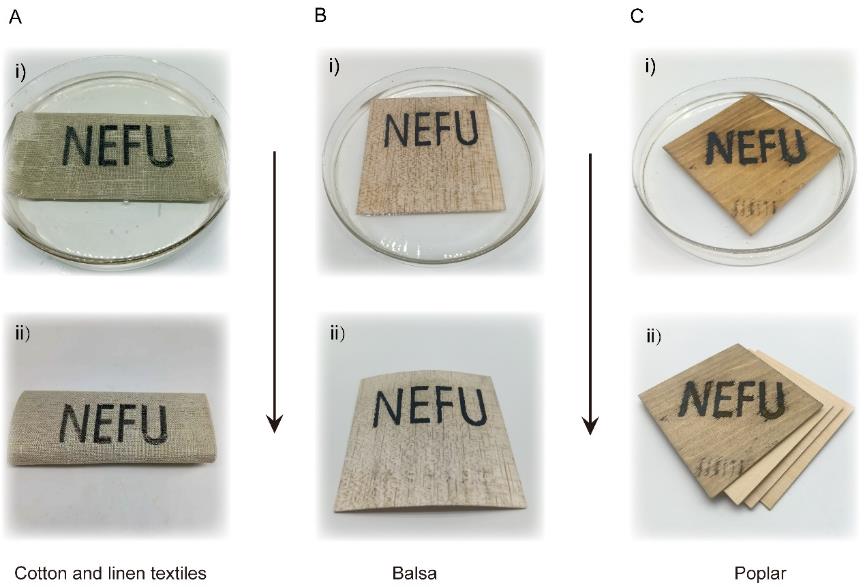


Fig. S14. Physical photos before (i) and after (ii) two days of immersion in water of patterning PEDOT on cotton linen (A), balsa wood (B) and poplar (C), respectively.


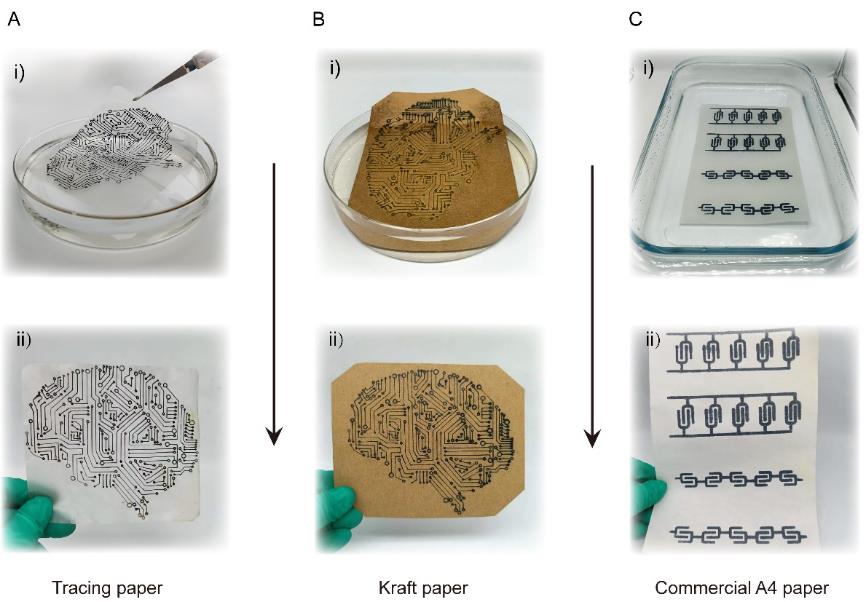


Fig. S15. Physical photos before (i) and after (ii) two days of immersion in water of patterning PEDOT on transfer paper (A), kraft paper (B) and filter paper (C), respectively.


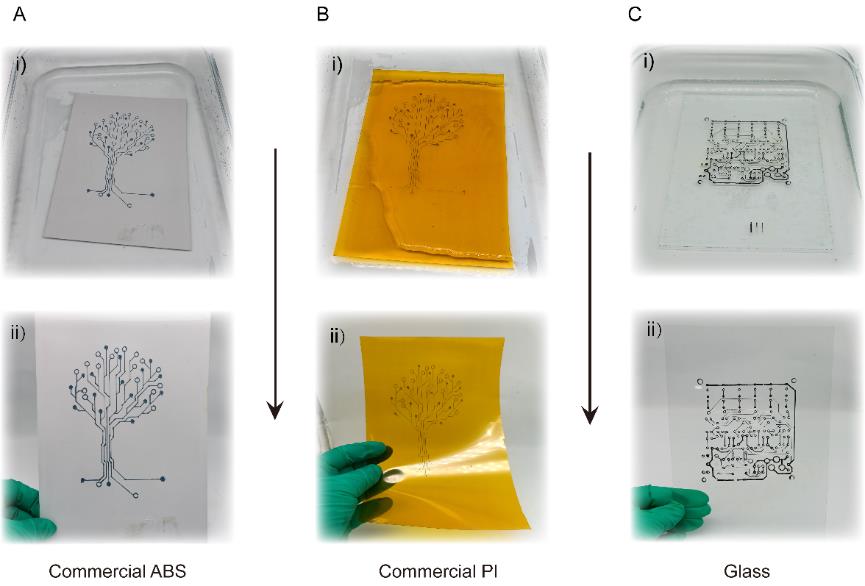


Fig. S16. Physical photos before (i) and after (ii) two days of immersion in water of patterning PEDOT on ABS (A), PI (B) and glass (C), respectively.


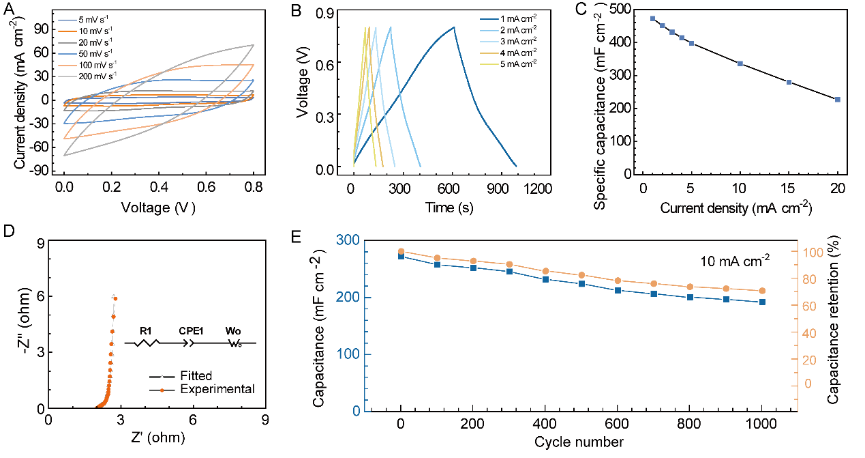


Fig. S17. Electrochemical performance of PEDOT-based sandwich flexible SC. A) CV curves at different scanning speeds. B) GCD profiles at different current densities. C) Plot of the area-specific capacitance calculated from the GCD profiles. D) Nyquist plots. E) Charge and discharge stability.

**
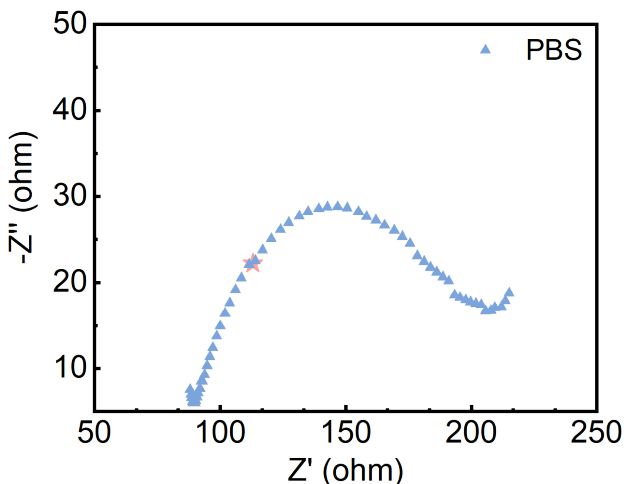
**

Fig. S18. Nyquist plot of a single channel of the printed neural electrode. The test range is 1 Hz-100000 Hz. The star point in the graph is labeled impedance at 1000 Hz. The area of the single electrode is 0.55cm^2^.


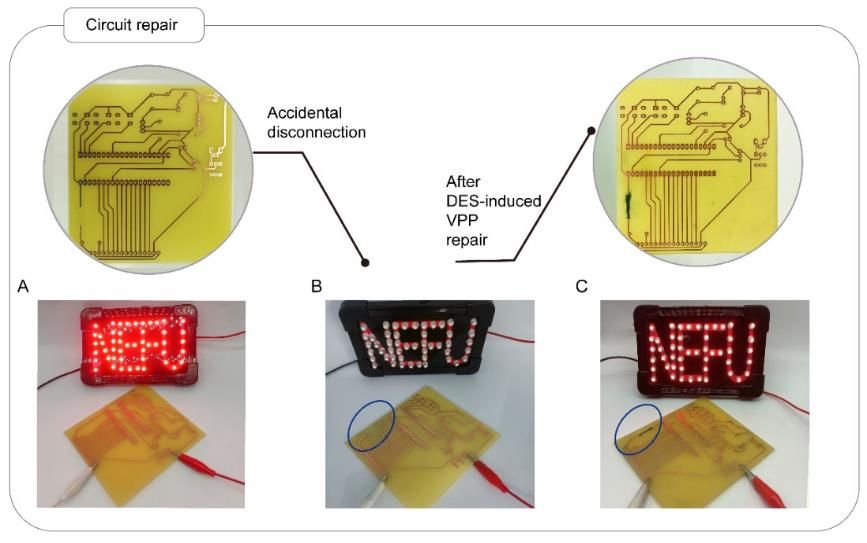


Fig. S19. Application demonstration of the DES-induced VPP method for circuit repair. Accidentally disconnected circuit boards can be re-energized in just 3 hours at 60 degrees. A-C) Physical photographs which showed energization of the original board (A), the board with an accidental break channel (B), and the conductive pathway in the circuit after the PEDOT repair (C), respectively.

Table S1: The technical indicators of the DES-induced VPP approach with conventional methods in Fig. 2B.

| Conductivity | Temperature | Interface robustness | Patternability | Material scalability | Methods |
| --- | --- | --- | --- | --- | --- |
| (S/cm) | (K) | (cycle) | (um) | (mm) |  |
| 240 | 353 | 50 | 1000 | 600 | Steam-assisted VPP |
| 0.14 | 313 | 100 | 10000 | 50 | Immersion-VPP |
| 0.05 | 303 | 200 | 10000 | 1200 | Roller-pressing |
| 0.576 | 393 | 100 | 10000 | 50 | Spin-coating |
| 2.82 | 313 | 500 | 500 | 500 | **This work** |

Table S2: Comparison of controlled PEDOT synthesis under time gradient.

| Time  (h) | Τ  (mm) | σ  (S m^-1^) | Ca*  (mF cm^-2^) | |
| --- | --- | --- | --- | --- |
| 0 | 0.175 | 0 | - | |
| 3 | 0.202 | 27.7 | 278.91 | ±21.96 |
| 6 | 0.207 | 65.8 | 588.01 | ±30.74 |
| 9 | 0.21 | 48.6 | 686.76 | ±39.15 |
| 12 | 0.216 | 90.6 | 1488.14 | ±70.28 |
| 15 | 0.241 | **282** | 1598.26 | ±102.63 |
| 18 | 0.255 | 268 | 1697.33 | ±196.42 |
| 21 | 0.26 | 183 | 1889.39 | ±305.43 |
| 24 | 0.262 | 163 | **2220.83** | **±307.52** |

*: current density at 1mA/cm^2^

Table S3: Comparison of power density and energy density of patterning PEDOT based flexible SC with other literature.

| Electrodes | Energy density  (μWh/cm^2^) | Power density  (μW/cm^2^) | Year |
| --- | --- | --- | --- |
| PEDOT: PSS/PVA/PMAA | 4 | 40 | 2020[38] |
| CNT + PEDOT: PSS/PAAM + SA | 3.6 | 200 | 2020[39] |
| PEDOT: PSS/PVA/PMAA | 0.65 | 170 | 2019[40] |
| PEDOT: PSS/PVA | 11.46 | 200 | 2021[41] |
| CMC-PEDOT/PAAM | 23.93 | 400 | 2022[37] |
| MXene/GNS/PPy@  PEDOT/Cotton | 322.15 | 460 | 2024[47] |
| PEDOT：PSS/rGO/MoS_2_ | 8.5 | 215.9 | 2023[48] |
| **Patterning PEDOT** | **42.02** | **400** | **This work** |
